# Supplementary figures and images for: Murine leukemia virus infection of non-dividing dendritic cells is dependent on nucleoporins
Source: PLoS Pathog. 2024 Jan 12;20(1):e1011640. doi: 10.1371/journal.ppat.1011640 (PMC10810426; doi:10.1371/journal.ppat.1011640)

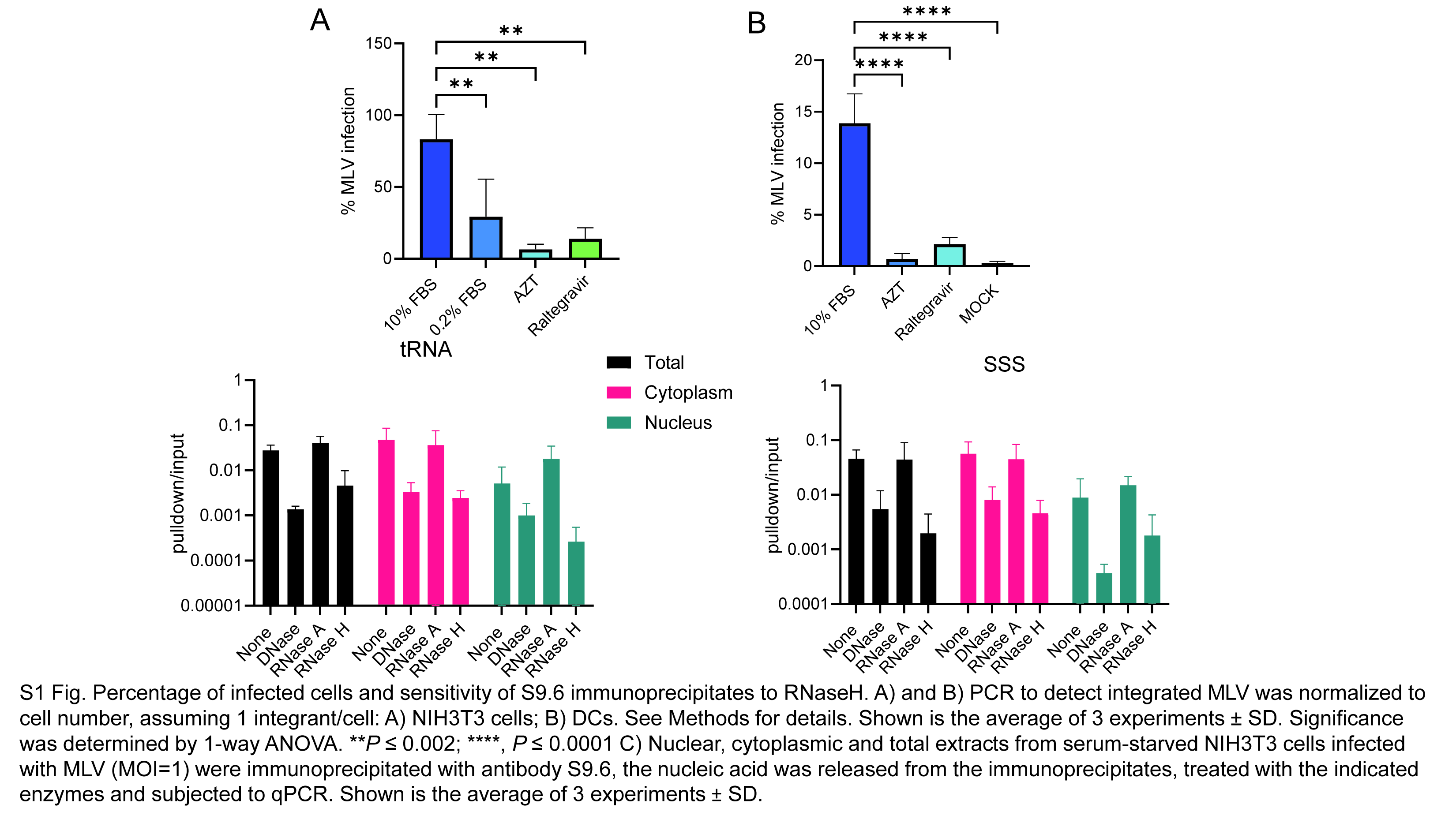

Supplement: S1 Fig — A) and B) PCR to detect integrated MLV was normalized to cell number, assuming 1 integrant/cell: A) NIH3T3 cells; B) DCs. See Methods for details. C) Nuclear, cytoplasmic and total extracts from serum-starved NIH3T3 cells infected with MLV (MOI = 1) were immunoprecipitated with antibody S9.6, the nucleic acid was released from the immunoprecipitates, treated with the indicated enzymes and subjected to qPCR. Shown is the average of 3 experiments ± SD. (TIF) [file ppat.1011640.s001.tif]

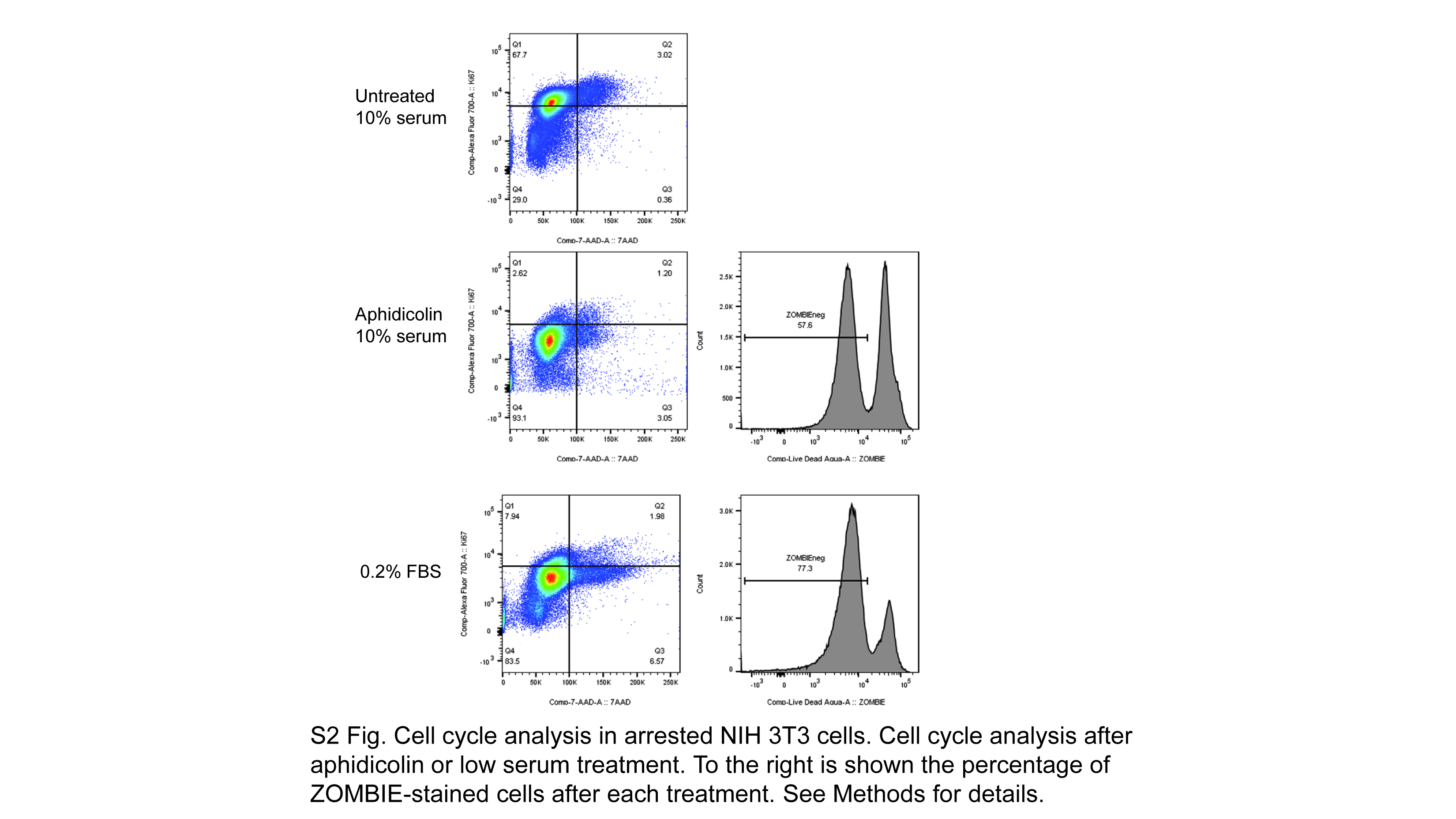

Supplement: S2 Fig — Cell cycle analysis after aphidicolin or low serum treatment. To the right is shown the percentage of ZOMBIE-stained cells after each treatment. See Methods for details. (TIF) [file ppat.1011640.s002.tif]

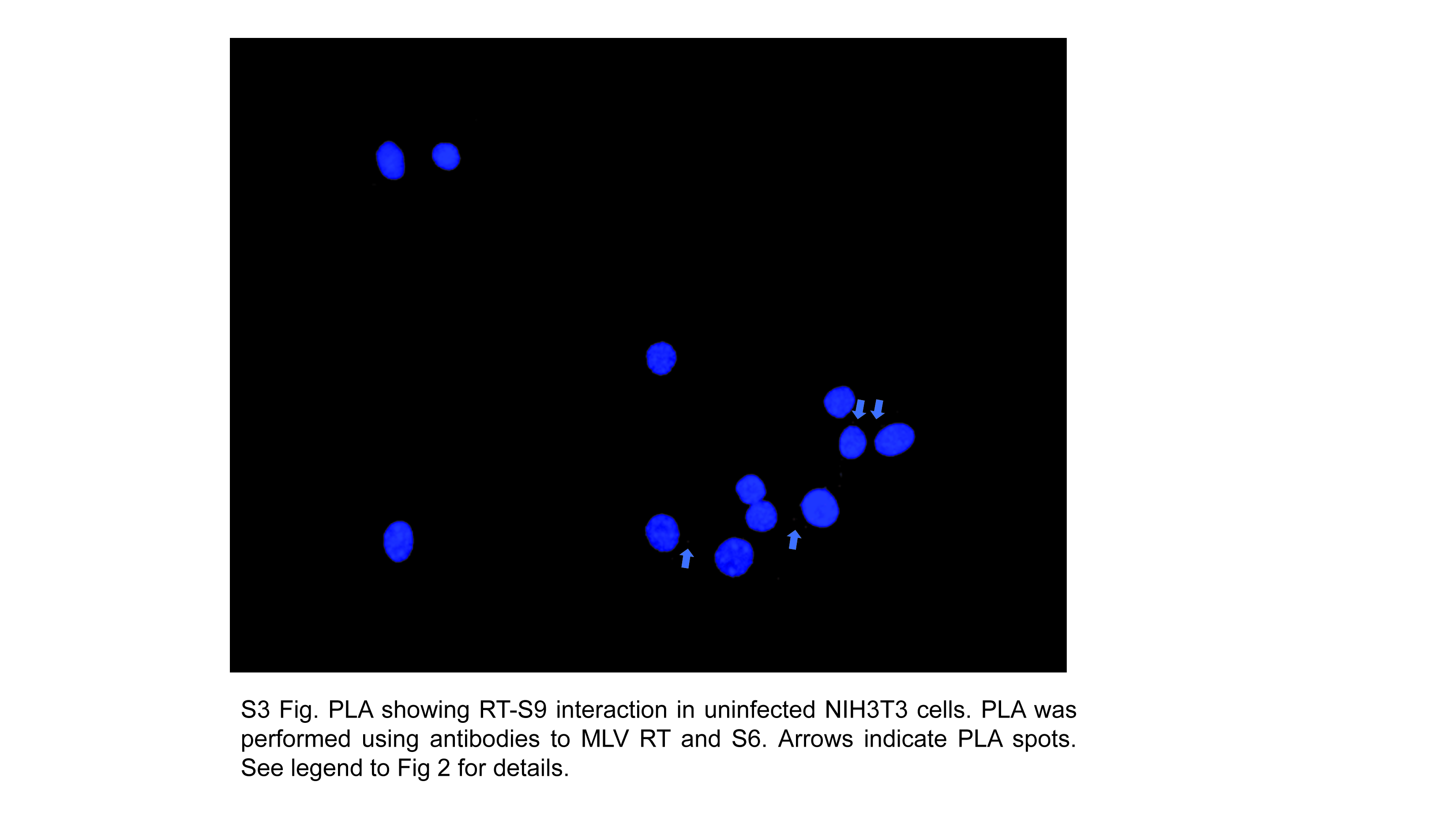

Supplement: S3 Fig — PLA was performed using antibodies to MLV RT and S9.6. Arrows indicate PLA spots. See legend to Fig 2 for details. (TIF) [file ppat.1011640.s003.tif]

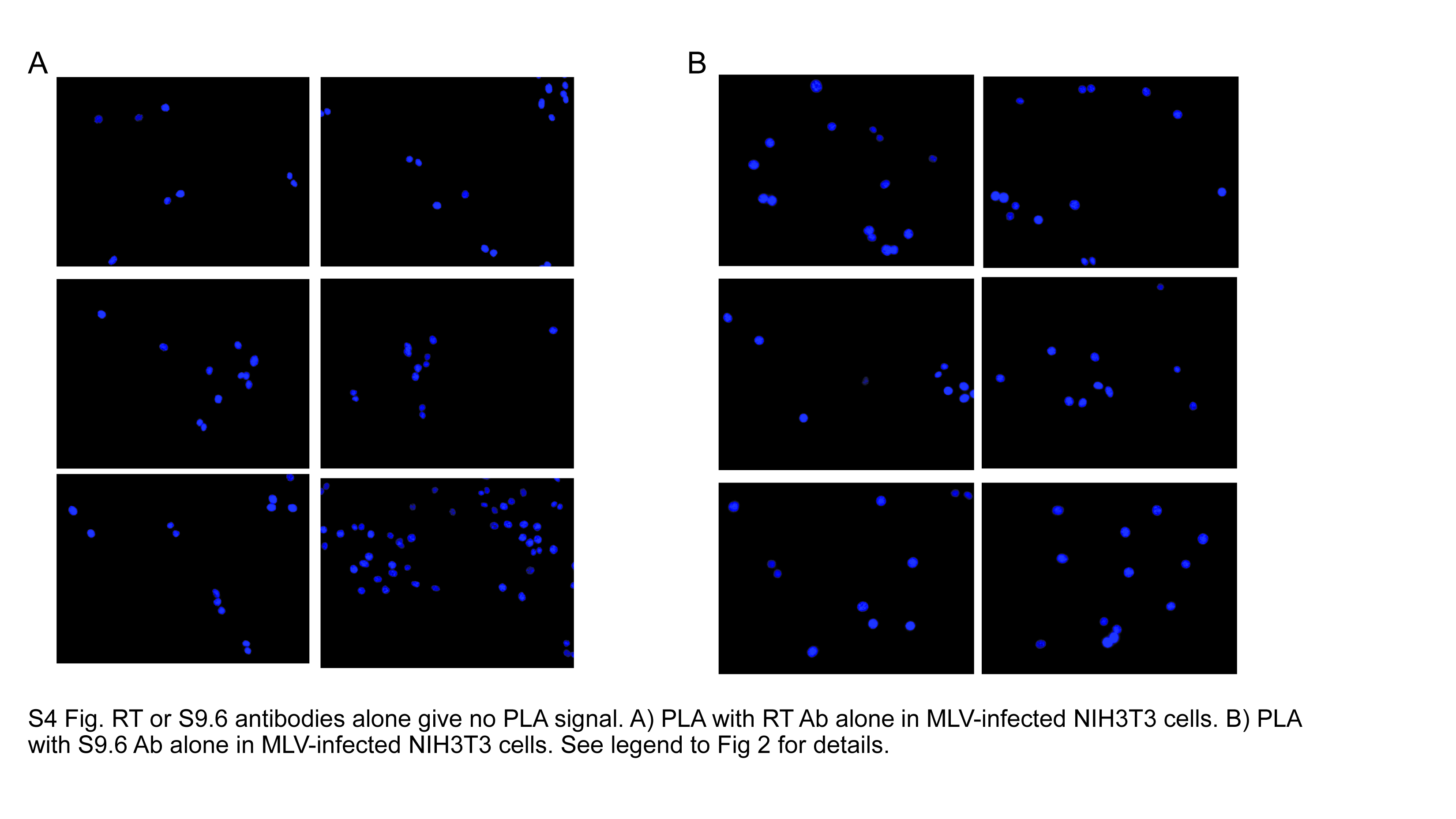

Supplement: S4 Fig — A) PLA with RT Ab alone in MLV-infected NIH3T3 cells. B) PLA with S9.6 Ab alone in MLV-infected NIH3T3 cells. See legend to Fig 2 for details. (TIF) [file ppat.1011640.s004.tif]

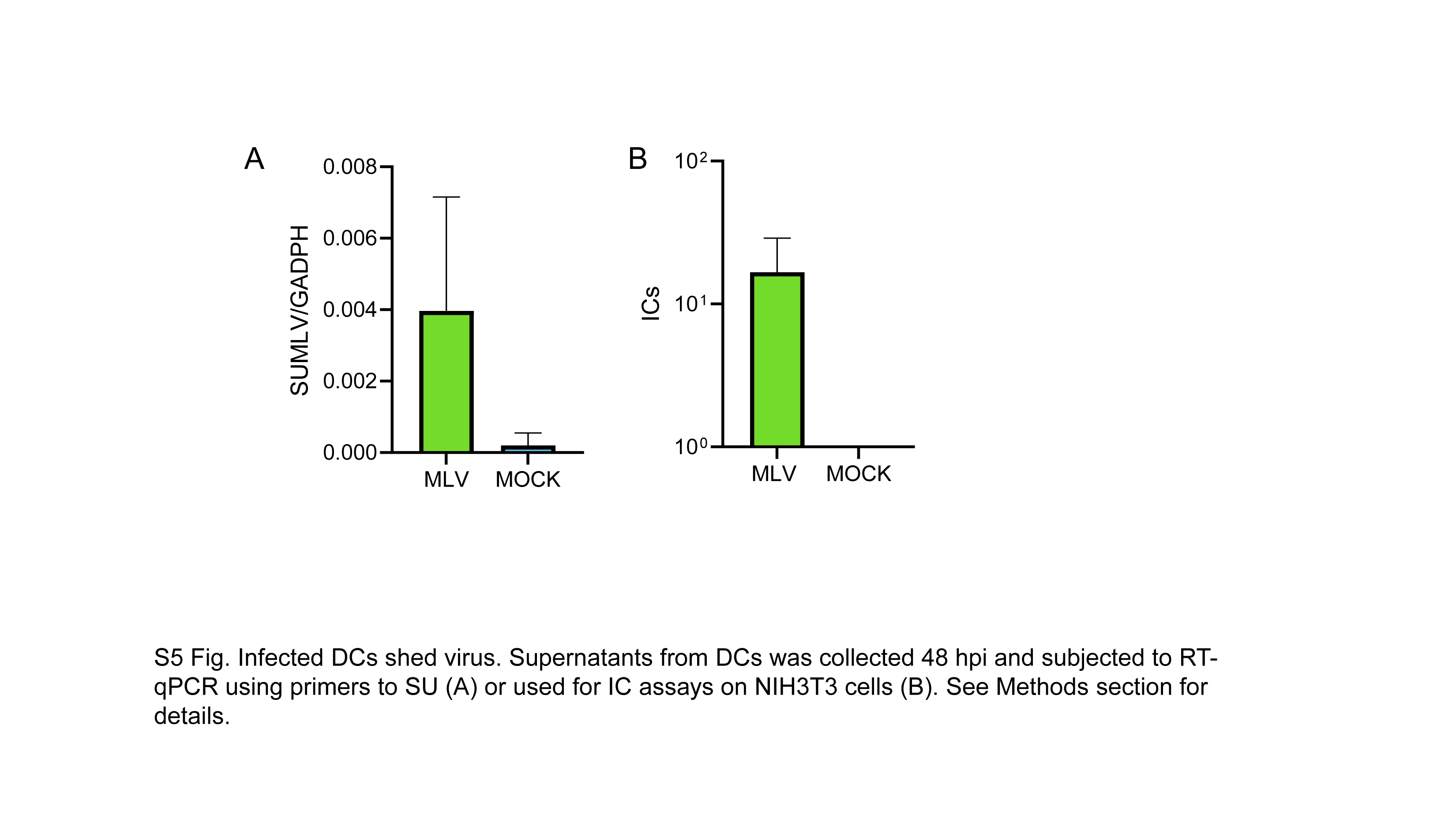

Supplement: S5 Fig — Supernatants from DCs was collected 48 hpi and subjected to RT-qPCR using primers to SU (A) or used for plaque assays on NIH3T3 cells (B). See Methods section for details. (TIF) [file ppat.1011640.s005.tif]

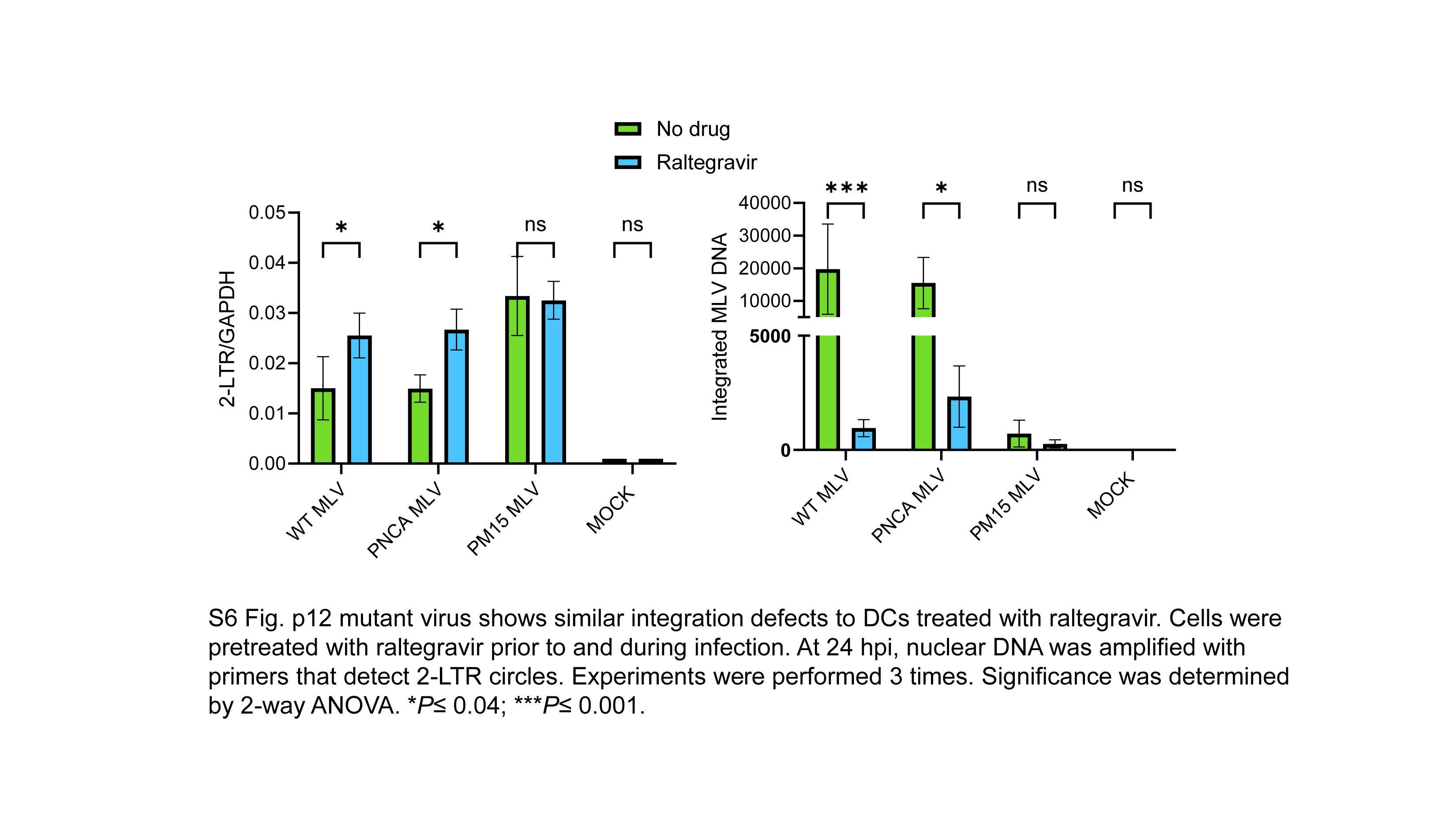

Supplement: S6 Fig — Cells were pretreated with raltegravir prior to and during infection. At 24 hpi, nuclear DNA was amplified with primers that detect 2-LTR circles. Experiments were performed 3 times. Significance was determined by 2-way ANOVA. *P≤ 0.04; ***P≤ 0.001. (TIF) [file ppat.1011640.s006.tif]

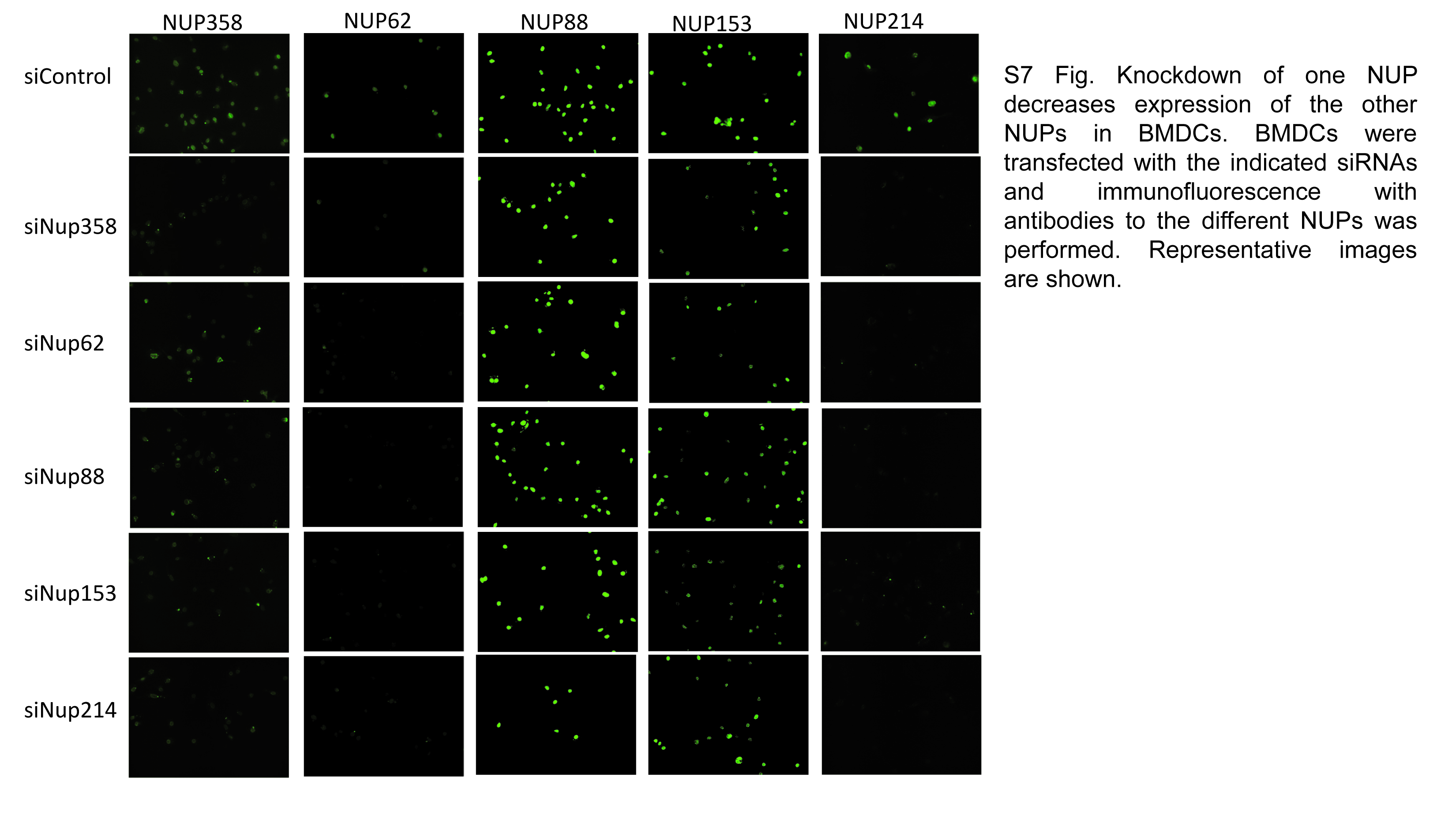

Supplement: S7 Fig — BMDCs were transfected with the indicated siRNAs and immunofluorescence with antibodies to the different NUPs was performed. Representative images are shown. (TIF) [file ppat.1011640.s007.tif]

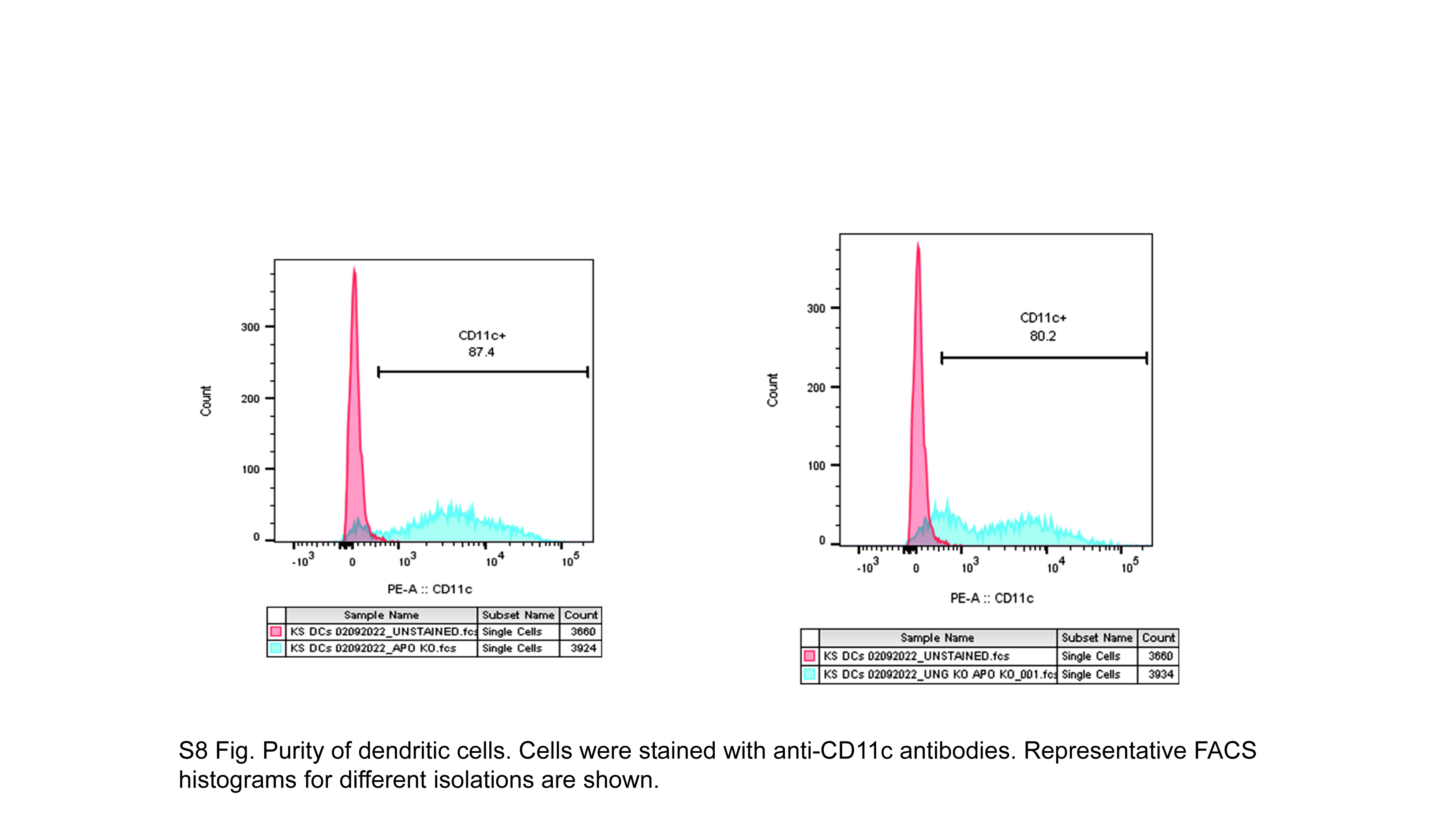

Supplement: S8 Fig — Cells were stained with anti-CD11c antibodies. Representative FACS histograms for different isolations are shown. (TIF) [file ppat.1011640.s008.tif]

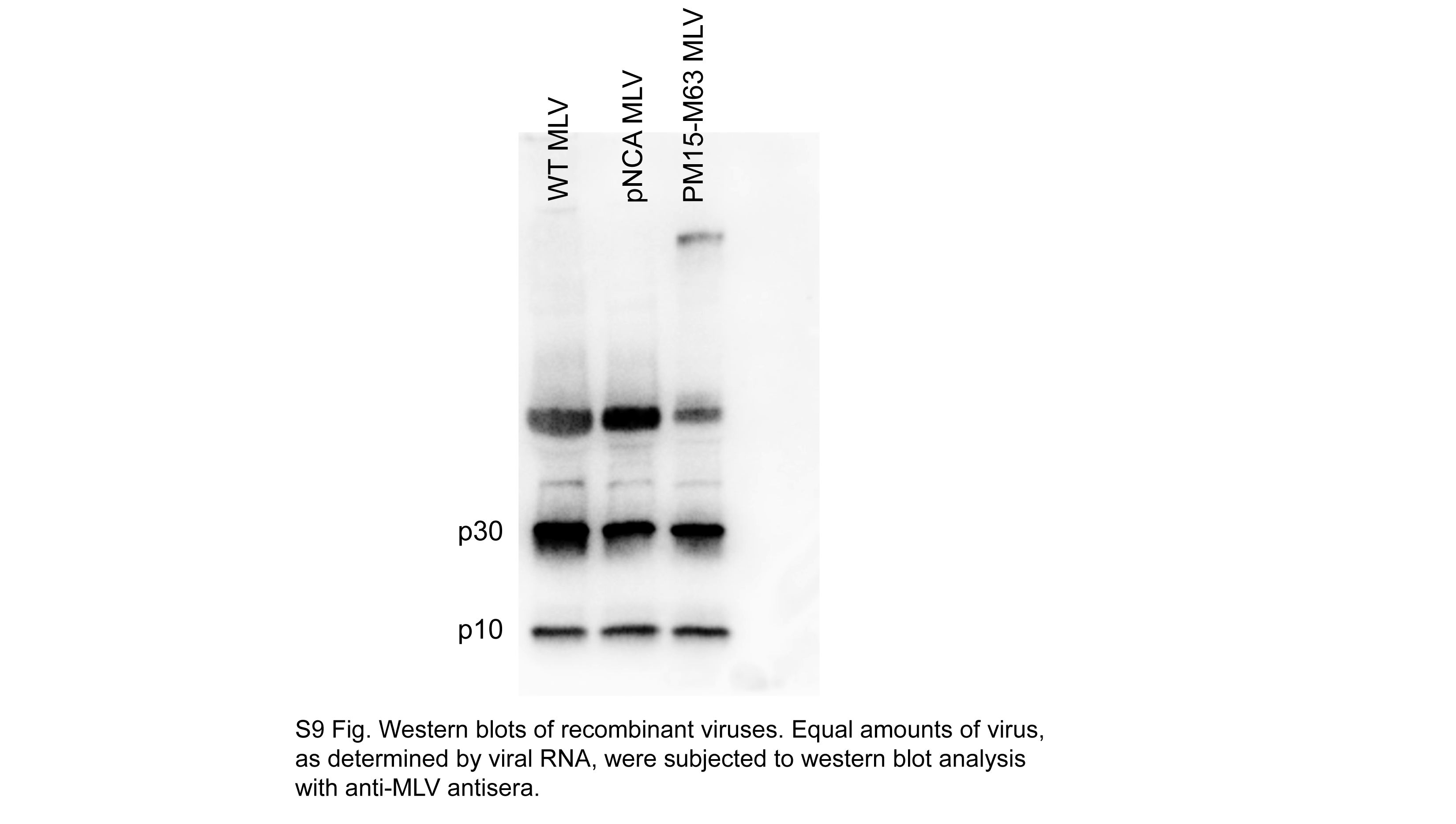

Supplement: S9 Fig — Equal amounts of virus, as determined by viral RNA, were subjected to western blot analysis with anti-MLV antisera. (TIF) [file ppat.1011640.s009.tif]
